# Learning process and improvement of point-of-care ultrasound technique for subxiphoid visualization of the inferior vena cava

**DOI:** 10.1186/s13089-016-0040-1

**Published:** 2016-03-31

**Authors:** Mauricio Gómez Betancourt, José Moreno-Montoya, Ana-María Barragán González, Juan Carlos Ovalle, Yury Forlan Bustos Martínez

**Affiliations:** Emergency Medicine Specialist, Universidad del Rosario, Bogotá, Colombia; Epidemiology, Biostatistics and Public Health Research Group, EPIBIOS_UR, Escuela de Medicina y Ciencias de la Salud (EMCS), Universidad del Rosario, Bogotá, Colombia; Chief of the Department of Emergency Medicine and Director of the Simulation Center, Escuela de Medicina y Ciencias de la Salud (EMCS), Universidad del Rosario, Bogotá, Colombia

**Keywords:** Learning curve, Ultrasonography, Inferior vena cava, Point-of-care, Residents, Colombia

## Abstract

**Background:**

Medical residents' training in ultrasonography usually follows the recommendations of the American College of Emergency Physicians (ACEP), even though these do not provide specific technical guidelines. Adequate training is considered to require 25 practical iterations in the majority of ultrasound procedures. However, the effectiveness of this approach has not been verified experimentally. We set out to determine the number of repetitions required for an acceptable ultrasound procedure of the inferior vena cava (IVC), as an important and emerging ultrasound procedure in cardiology.

**Methods:**

Using three human models, each of eight medical residents in the Emergency Medicine (EM) Program at the Universidad del Rosario performed 25 iterations of the recommended procedure, with image quality evaluation by an EM physician expert in the technique. Logistic regression analysis was used to determine the lowest number of repetitions required to achieve an adjusted probability of success of 80 and 90 %, respectively.

**Results:**

We obtained 200 ultrasound images. The percentage success by each resident ranged from 52 to 96 %. There was no statistical significance in the relation between gender and success (*p* = 0.83), but there was an association between year of residency and success (*p* < 0.001). The average time taken for each procedure was 17.3 s (SD 8.1); there was no association between the time taken and either repetition number or image quality. We demonstrate that eleven repetitions are required to achieve acceptable image quality in 80 %, and that 21 repetitions are required to achieve acceptable image quality in 90 %.

**Conclusions:**

This is the first study to formally evaluate the effectiveness of recommended training in ultrasound techniques. Our findings demonstrate that training comprising 25 procedural repetitions is easily sufficient to achieve optimal image quality, and they also provide empiric knowledge toward elucidating the times and minimum repetitions needed to acquire and improve ultrasonographic technique in novice operators to a level which fulfills quality requirements for interpretation.

## Background

Emergency Ultrasound, also known as “point-of-care” or “bedside ultrasound,” is part of the essential training requirement for Emergency Medicine (EM) and intensive care programs in the United States and elsewhere [1–3]. Cardiac focused ultrasound is part of the compulsory recommendations of physician training in critical care. The assessment of the IVC, which is part of this cardiac focused study, has been gaining importance due to its implications for the management of patients in shock [4–8]. In Colombia, the use of ultrasonography by specialized physicians is now in its initial phase, because of the recent inclusion of this training in the curriculum of EM programs. In addition, against the massive adoption of this practice arises the limited availability of equipment in emergency services and competent operators [2, 9]. Early diagnosis and treatment of critical care patients in the developed and developing world is supported by the diagnostic performance of the point-of-care ultrasound technique for subxiphoid visualization of the IVC, which has been reported to be superior to invasive tools such as central venous pressure, and similar to arterial pressure curve methods [10–12].

The American College of Emergency Physicians (ACEP) recommends a minimum of 25 repetitions for developing competence in ultrasonography. The aforementioned was based on learning curves for different techniques and approach windows, as well as expert recommendations [1]. However, the guidelines are not specific for the subxiphoid visualization of the IVC, and the number and characteristics of the required studies for optimal performance are still unknown [11]. Available literature regarding the learning of ultrasonographic techniques, such as that reported by Gaddis et al., suggests that the accuracy of Focused Abdominal Sonography for Trauma (FAST) increases with repetitive experience [13]. Consequently, the appropriation of the procedure not only will be useful for its application as a monitoring and evaluation tool of effectiveness of novice physician’s training programs in ultrasonography, but also as a practical device in diagnosis schemes. This paper was aimed to assess the learning and improvement process of point-of-care ultrasound technique for subxiphoid visualization of the IVC in EM residents according to the ACEP recommendations.

## Methods

### Population and operators

Students from all years’ EM residency participated as operators in this study once they attended a 40-min conference by an expert in ultrasound on basic principles and techniques for performing IVC ultrasonography visualization through subxiphoid window. Operators with previous training in ultrasonography were excluded. We used three models for the simulation of the procedure considering healthy adult males, aged 29, 33, and 39, with BMI of 26.8, 18, and 25, respectively. The study was conducted at the Center for Clinical Simulation at the Universidad del Rosario in Bogota, Colombia. All residents and participants signed informed consent, and the protocol was approved by the ethics committee of the same university.

### Technique

The equipment used was the *Sonosite ultrasound*-EDGE C60 with a 2–5 MHz transducer [14]. Transducer was positioned in the epigastrium and directed toward the left shoulder where, with the indicator on the right, the cardiac view was obtained. Then, the transducer was rotated 90° until the indicator was cephalic, where the longitudinal view of the IVC was obtained. At this point, a six-second video was taken.

Success was defined as a score of three which, according to the ACEP guidelines [1], allows the minimum adequate identification of key structures needed to make clinical decisions; clear identification of the inferior vena cava with visualization of at least 2 cm distal to the entrance of the vena cava to the right atrium, where the measurement of a cross-sectional cut should be performed. It was not necessary for the images to be centered, nor to have the adequate image depth.

### Experimental design

We used a complete factorial experimental design, i.e., a set of all possible combinations between models and operators [15]. The sample size of the experiment was set at 25 repetitions per operator, according to the recommendations of the guidelines for emergency ultrasonography, and, in order to ensure the minimum number of observations per subject [16]. This type of experiment allows to examine simultaneously the effect of each level of observation (repetition and residents) on the dependent variable, as well as the effect of interactions among factors. Data collection was conducted in March, 2015.

Variables included year of residency, gender, and the duration of each repetition defined as the time in seconds elapsed from when the transducer touches the skin and the operator determines that the best image of the IVC through the subxiphoid window has been obtained, until the end of a 6 s recording of the image.

All data were stored electronically through anonymous records and were evaluated by the expert, using the scale for quality assurance proposed by the ACEP. Table 1 shows the ACEP suggested quality assurance grading scale, 2011 [17].

**Table 1:** Suggested quality assurance grading scale ACEP, 2011

Punctuation	Definition
1	No recognizable structures, no objective data can be gathered
2	Minimally recognizable structures but insufficient for diagnosis
3	Minimal criteria met for diagnosis, recognizable structures but with some technical or other flaws
4	Minimal criteria met for diagnosis, all structures imaged well and diagnosis easily supported
5	Minimal criteria met for diagnosis, all structures imaged with excellent image quality and diagnosis completely supported

The scale consists of five levels. *1* is the lowest grade: image fails to recognize anatomical structures and therefore does not allow a diagnosis. *2* recognizes anatomical structures but not enough to make a diagnosis. *3* image allows a diagnosis, but there are certain failures in the image’s position or image gain and depth. *4* and *5*, diagnostic is supported by images of good and excellent quality, respectively

### Statistical analysis

The descriptive analysis of quantitative variables was carried out by calculating means and standard deviations (SD), whereas categorical variables were reported as frequencies and percentages. The homogeneity of the answers by subgroups conformed by residents, gender, and year of residency was assessed by means of χ^2^-like tests; the association between the time and the number of subxiphoid window ultrasound examinations was evaluated using Spearman’s rank correlation coefficient [18]. To identify the learning curve, we developed a two-level repeated measures model [19, 20], considering the residents as clustering units. The model (with success/failure variable response) was based on the logistic function to estimate the adjusted probabilities of success [19, 20]. All analyses were performed using the STATA V.12 statistical package [21].

## Results

We collected information on eight operators: three first year, two second year, and three third year residents. We did not collect information on three more ones (two first year and one third year) because they had previous training in ultrasonography. The final four residents enrolled in the program left the experiment voluntarily. The sample size reached 200 repetitions.

The most of repetitions (*n* = 114) were graded as three [11]. Twenty-two repetitions obtained the highest score (11 %), and nine repetitions earned the minimum score. The percentage of success by resident was between 52 and 96 % (Table 2). No relationship was found between gender and success (*p* = 0.83), but there was a relationship between year of residency and success (*p* < 0.001).

**Table 2:** Percentages and mean time for subxiphoid visualization of the IVC by novice EM Colombian residents

Resident	Success^a^		Time (seconds)	
	*n*	%	Mean	SD
1	20	80	13.5	4.1
2	19	76	16.7	8.2
3	23	92	24.1	9.6
4	13	52	17.6	9.8
5	17	64	17.2	5.44
6	24	96	15.7	7.2
7	22	88	18.9	9.3
8	24	96	14.7	4.3
Total	200	81	17.3	8.06

First column represents the total of residents that participate in the experiment. Followed columns represent the number and the percentage of success defined as a grading equal or greater than 3 according to an expert fulfillment of ACEP criteria [16]. Finally, the table presents mean time and standard deviations in seconds used by each resident in performing the repetitions

^a^Expert grading equal or greater than 3, according to ACEP [16]

The average total time per repetition was 17.3 s (SD 8.1) (Table 2), although there was no statistically significant association between length of the repetition and the number of repetitions (*p* = 0.18). In addition, no significant differences were found between length of repetition and outcome (success/fail) (*p* = 0.2).

**Fig. 1:**
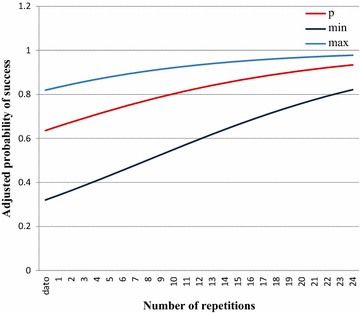
Learning curve for point-of-care ultrasound technique for subxiphoid visualization of the IVC by novice EM Colombian residents. The *y axis* shows the probability of success adjusted by repetition and resident. The *x axis* shows the repetition number. The *curve* shows how the probability of success increases with number of repetitions. The *red line* is the average probability, while the *blue lines* represent minimum and maximum values

Regarding the learning curve, we calculated the probability of success adjusting by repetition and resident (Fig. 1); we estimated a maximum probability of 0.8 for repetition 11 (range 0.54 a 0.92), and repetition 21 had a probability of 0.9 (range 0.75–0.96).

## Discussion

This work assessed the learning process and the improvement in proficiency of the point-of-care ultrasound technique in Colombian EM residents. For that, we built a learning curve in order to guarantee a proportion of success greater than 80 % in performing the point-of-care ultrasound technique for subxiphoid visualization of the IVC, with adequate quality according to the ACEP, in novice EM residents. The number of repetitions needed to achieve 80 % success was found to be eleven, and for 90 % success, twenty-one repetitions were needed. No differences were found between success percentages by gender, but there were differences by year of residency. However, due to the low number of residents, the multivariate model was not adjusted by year of residency. No statistically significant differences were found related to the time needed to acquire the image.

These results support the hypothesis that fewer than twenty-five repetitions are needed to achieve an acceptable quality ultrasonographic image of the IVC which is useful for decision making in clinical practice [17]. Previous evidence reported heterogeneous results about the number of repetitions [22–24] due to methodological or context variations of each scenario. Some authors have reported 10 repetitions as the optimal number needed to visualize anatomic structures in the right upper abdominal quadrant [25].

Our results should be interpreted with caution to the extent that the small sample size leads to a model with precision limitations in the effect measurement. However, the big percentages of success with increased numbers of repetitions allowed us to think there would be similar results with bigger sample sizes. The number of models was chosen based on practical issues and the ACEP recommendations to assess the quality of the ultrasound images, even without being validated. In consequence, future research must include an extensive group of individuals, local models, and operators.

Accordingly, the scope of the results is in some way limited to our own background. As far as we know, this is the first study in Colombia designed for building a learning curve for this technique, and controlled by the operators’ adaptation. Moreover, in so far as the agreement between residents and the expert regarding the perception of the best ultrasound window was partially assessed, finding a measure of collapsibility or vena cava diameter may require a similar number of repetitions. In this regard, Fields et al. suggested at least 15 repetitions in order novice operators to obtain a reliable measure of IVC diameter through the subxiphoid ultrasound window. This result was not generated from a curve, but based on the assumption that an additional five repetitions above the baseline of 10 repetitions increases the degree of congruence between the residents and the experts [26].

Finally, although these results correspond to the practical verification of the average time used to improve the technique of IVC ultrasound window in the Colombian context, they set a precedent for the evaluation of this type of diagnostic procedures in the local and regional community. Lastly, methodology used makes it easy to extrapolate the evaluation process carried out here to other areas of medical education assessment, particularly with regard to obtaining other, more complex, good quality ultrasound windows.
